# Has the Prevalence of Migraine Changed over the Last Decade (2003–2012)? A Spanish Population-Based Survey

**DOI:** 10.1371/journal.pone.0110530

**Published:** 2014-10-24

**Authors:** César Fernández-de-las-Peñas, Domingo Palacios-Ceña, Jaime Salom-Moreno, Ana López-de-Andres, Valentín Hernández-Barrera, Isabel Jiménez-Trujillo, Rodrigo Jiménez-García, Carmen Gallardo-Pino, María S. García-Gómez-de-las-Heras, Pilar Carrasco-Garrido

**Affiliations:** 1 Department of Physical Therapy, Occupational Therapy, Rehabilitation and Physical Medicine, Universidad Rey Juan Carlos, Alcorcón, Spain; 2 Esthesiology Laboratory, Universidad Rey Juan Carlos, Alcorcón, Spain; 3 Department of Preventive Medicine and Public Health, Universidad Rey Juan Carlos, Alcorcón, Spain; 4 Department of Human Histology, Universidad Rey Juan Carlos, Alcorcón, Spain; Medical Sciences Campus, Puerto Rico

## Abstract

**Introduction:**

Information on temporal trends can identify groups of people at risk for any particular condition; however information on temporal trends on migraine headache at population levels is scarce. Our aim was to estimate the time trends in the prevalence of migraine from 2003 to 2012 in Spain.

**Methods:**

A population-based national study was conducted. We analyzed data using individualized information taken from national surveys conducted in 2003/4, 2006/7, 2009/10 and 2011/12. A total of 94,158 Spanish adults participated. We considered the presence of self-rated and diagnosed migraine, and we analyzed socio-demographic features, lifestyle habits, self-rated health status, and comorbid diseases using logistic regressions.

**Results:**

The prevalence of migraine increased from 6.54% in 2003 to 9.69% in 2012 with significant time trends (adj. OR 1.65; 95%CI 1.50–1.81). The probability of women of suffering migraine was 3 times higher than for men (adj.OR 3.08; 2.82–3.37). There was a declining trend in migraine prevalence as age increased (adj.OR 0.42; 0.35–0.51). Demographic variables associated with migraine were lower educational level (adj.OR 1.32; 1.13–1.54) and not being an immigrant (adj.OR 1.37; 1.15–1.64). A worse self-reported health status was related to higher prevalence of migraine (adj.OR 2.83; 2.59–3.09). The prevalence of migraine also increased as the number of comorbid conditions increased (adj.OR 2.42; 2.05–2.86).

**Conclusion:**

The prevalence of migraine has increased in the first decade of the 21^st^ century in Spain. Migraine was associated with being female, mid-age, low educational level, not being an immigrant, worse self-rated health status and presence of comorbid conditions.

## Introduction

Headache is probably the most prevalent neurological disorder seen by medical doctors and usually experienced by almost everyone at some moment during their lives. Migraine is one of the most common headaches and may cause substantial disability for patients and their families as well as to the global society due to very high prevalence in the general population [1].

Several studies have estimated the annual point-prevalence of migraine in several countries [2]–[6]. In general, the average lifetime prevalence of migraine seems to be of 18%, whereas the estimated average one-year prevalence is around 13% [7], [8]. However, because of the economic and health impact of migraine, knowledge of point prevalence is not enough for promoting health programs in Public Health Systems. Information on temporal trend can help to identify groups of people at risk for any particular condition, evaluate public health interventions, and help to develop specific preventive actuations.

Nevertheless, information on temporal trends in migraine at population levels is scarce. Some studies observed an increasing temporal trend in the incidence of migraine in the last decade of the twentieth century in both child-adolescents [9] and adults [10]. Two studies analysing time trends of migraine prevalence in adults from the last decade of the 20^th^ century reported that the prevalence of migraine remained stable [11], [12]; although in the latter study, a significant increase in the proportion of individuals with frequent migraine was observed [12]. A recent study conducted in Norway found an increase in the prevalence of migraine from 12.1% to 13.2% during an 11-year period [13].

Taken together, these data suggest that further studies are needed. In fact, most of the published studies investigated temporal trends in the last decade of the 20^th^ century [9]–[12], with only one including some years of the 21^st^ century [13]. To the best of the author's knowledge, no published study has previously investigated time trends of the prevalence of migraine in Spain. The current study examines the time trends in the prevalence of migraine for Spanish adults using National Health Survey conducted in the first decade of the 21^st^ century. Therefore, the main aim of the current study was to analyze the time trend in the prevalence of migraine in the period from 2003 to 2012. We also analyzed the association of migraine with socio-demographic factors, lifestyle habits, self-reported health status, and co-morbidity with other chronic diseases.

## Methods

### Spanish National Health Surveys (SNHS) - European Health Interview Survey for Spain (EHSS)

We conducted a cross-sectional study using individualized data taken from those national surveys conducted in 2003/4 (SNHS), 2006/7 (SNHS), 2009/10 (EHSS), and 2011/12 (SNHS). All the surveys included in this study were conducted by the National Statistics Institute (*Instituto Nacional de Estadística, INE*) under the aegis of the Spanish Ministry of Health & Consumer Affairs using an almost identical methodology.

All surveys are based on ongoing, home-based personal interviews examining a nation-wide representative sample of non-institutionalized civilians residing in main family dwellings (households) of Spain. Adults were defined as those subjects aged ≥16 years in the 2003–2006 SNHS and in the 2009 EHSS, and those aged ≥15 in the 2011 SNHS. In the current study, we considered adults those subjects aged ≥16 years in all the surveys. Participants were selected by probabilistic multistage cluster sampling with proportional random selection of primary and secondary sampling units (towns and sections, respectively) with the final units (subjects) being selected by means of random routes and sex- and age-based quotas. Surveyors were trained on basic communication skills, procedures and the questionnaires. In order to meet the surveys' stated aim of being able to furnish estimates with a certain degree of reliability at both national and regional levels the following data collections periods and samples of adults were selected: 2003 SNHS (data collection: April 2003–March 2004; sample: 21,650 subjects), 2006 SNHS (data collection: July 2006–June 2007; sample: 29,436 subjects), 2009 EHSS (data collection: April 2009–March 2010; sample: 22,188 subjects), 2011 SNHS (data collection: July 2011–June 2012; sample: 21,007 subjects). Details on the surveys methodology are described elsewhere [14]–[17].

### Study variables

The variables included in our study were based on a series of questions included identically in all the surveys. Individuals were classified as *migraine sufferers* if they responded “yes” to both of the following questions: “Have you suffered migraine over the previous 12 months?” AND “Has your neurologist confirmed the diagnosis?”.

We analyzed socio-demographic characteristics (i.e. gender, age, marital status, educational level, occupation status, and migration status); self-perceived health status; lifestyle-related habits (i.e., smoking habits, alcohol consumption, sleeping habits, physical exercise, and obesity); and the presence of diagnosed comorbid diseases or symptoms including heart disease, high blood pressure, arthritis, asthma, bronchitis, diabetes mellitus, osteoporosis, menopausal symptoms, and depression as independent variables. The number of chronic conditions was categorized as “none”, “one or two” and “three or more”.

Within socio-demographic characteristics, educational level was classified into “no studies”, “primary”, “secondary” or “university studies”; whereas the occupation status was reflected as “unemployed”, “employed”, or “retired”. Self-perceived health status was assessed with the following question: “How did you perceive your health status over the last 12 months?” Subjects rated their health status as “excellent”, “good”, “fair”, “poor”, or “very poor”. This variable was dichotomized into “excellent/good” or “fair/poor/very poor” self-perceived health status.

Within lifestyle habits, smoking habit differentiated between current smokers, ex-smokers or non-smokers. The alcohol consumption was measured using the question “Have you consumed any alcoholic drink in the last 2 weeks?” Sleep habit was divided into subjects who sleep ≥8 hours/day and those sleeping <8 hours/day (this variable was not collected in the 2009 EHSS). Subjects were also asked for: “Do you practice leisure time moderate or intense physical activity for at least 3 days per week during your free time?” Finally, the body mass index (BMI) was calculated from the self-reported body weight and height. Individuals with a BMI ≥30 were classified as obese.

### Statistical analysis

We first estimated the crude prevalence of Spanish adults with their associated 95% confidence interval (CI) who were classified as migraine sufferers for each survey and described their distribution according to the study variables. Secondly, we analyzed the association of these variables with the presence of migraine using chi square tests. Thirdly, multivariate unconditional logistic regression models were generated, so we could determine which of the variables were associated with suffering migraine in our population. In this model, we used the year of the survey (2003 as a reference) to assess the time trend after controlling for the remaining independent variables (sleeping hours was excluded since it was not included in one survey). Three multivariate models were conducted one for each gender and other for the whole population in order to evaluate the effect of gender.

Estimates were conducted using the "*svy*" (survey commands) functions of the STATA program, which enabled us to incorporate the sampling design and weights into all statistical calculations (descriptive OR, logistic regression). Statistical tests were considered significant when the P value was less than 0.05.

## Results

### Baseline demographics

The total number of subjects aged ≥16 years interviewed in all the surveys was 94,158. The distribution by socio-demographic variables of the adults included in the study is shown within **Table 1**. The study weighted populations included slightly more women than men (51% vs. 49%) and the mean age slightly increased from 50.1 years to 51.8 years old along the time period (P<0.05).

**Table 1:** Distribution by socio-demographic variables of the adults included in the national health surveys conducted in Spain from 2003 to 2011.

VARIABLE	CATEGORIES	2003 SNHS (N = 21650) N (%*)	2006 SNHS (N = 29436) N (%*)	2009EHSS (N = 22188) N(%*)	2011 SNHS (N = 20884) N (%*)
**Sex**	Male	9,875 (48.8)	11,645 (49.1)	10,045 (49.0)	9,579 (48.8)
	Female	11,775 (51.2)	17,833 (50.9)	12,143 (51.0)	11,305 (51.2)
**Age#**	Mean (SD)	50.1 (19.3)	50.6 (18.6)	51.2 (18.8)	51.8 (18.9)
	16–30 years	3,902 (26.6)	4,536 (24.1)	3,248 (22.7)	2,934 (20.6)
	31–50 years	7,745 (36.5)	11,170 (38.4)	8,222 (38.6)	7,520 (39.0)
	51–70 years	5,858 (24.4)	8,198 (24.4)	6,381 (25.1)	6,252 (26.5)
	>70 years	4,145 (12.6)	5,574 (13.2)	4,337 (13.5)	4,178 (13.9)
**Marital status**	Unmarried	5,947 (33.0)	7,425 (31.7)	5,886 (31.4)	5,787 (31.5)
	Married	12,153 (57.1)	16,731 (57.1)	12,009 (56.5)	10,979 (56.3)
	Widow	2,788 (7.3)	3,672 (7.1)	2,893 (7.6)	2,746 (7.4)
	Separated/divorced	762 (2.6)	1,583 (4.1)	1,388 (4.5)	1,351 (4.8)
**Educational level**	University	2,894 (14.6)	4,452 (17.0)	3,688 (17.0)	3,138 (15.9)
	Secondary	3,923 (19.2)	7,222 (28.3)	5,598 (28.0)	5,421 (28.3)
	Primary	11,549 (52.1)	13,581 (43.4)	8,992 (40.6)	9,258 (44.2)
	No studies	3,284 (14.1)	4,086 (11.3)	3,888 (14.3)	3,067 (11.5)
**Occupational status**	Employed	12,254 (63.5)	13,624 (51.4)	11,336 (55.6)	8,738 (44.9)
	Unemployed#	744 (3.8)	1,840 (7.1)	2,187 (11.6)	2,630 (14.6)
	Retired	8,652 (32.7)	14,014 (41.5)	8,655 (32.9)	9,516 (40.5)
**Immigrant**	Yes#	650 (3.7)	1,859 (10.7)	1,371 (12.4)	1,321 (12.0)
	No	21,000 (96.3)	27,577 (89.3)	20,817 (87.6)	19,563 (88.0)

* Weighted percentage/# Statistically significant increasing with time (P<0.005).

### Prevalence of migraine: bivariate analysis

A total of 9,357 adults (9.94%) answered affirmatively to both questions about migraine. The prevalence of migraine increased from 6.54% in 2003 to 9.69% in 2012 with a significant unadjusted time trend (P<0.01). The prevalence of migraine according to socio-demographic variables is summarized in **Table 2** **.**

**Table 2:** Prevalence of migraine by socio-demographic variables among Spanish adults, according to the national health surveys conducted in Spain from 2003 to 2012.

VARIABLE	CATEGORIES	2003 SNHS % (95%CI)	2006 SNHS % (95%CI)	2009 EHSS % (95%CI)	2011 SNHS % (95%CI)
**Sex^abcd^**	Male	3.95 (3.48–4.48)	6.51 (5.92–7.15)	5.39 (4.89–5.95)	4.91 (4.42–5.45)
	Female#	9.02 (8.39–9.69)	17.16 (16.42–17.93)	15.98 (15.21–16.78)	14.24 (13.48–15.04)
**Age^abcd^**	16–30 years	4.26 (3.57–5.08)	9.33 (8.28–10.50)	7.95 (6.95–9.07)	7.15 (6.17–8.27)
	31–50 years	6.46 (5.82–7.18)	12.75 (11.97–13.57)	10.68 (9.91–11.50)	9.75 (8.99–10.56)
	51–70 years	8.63 (7.77–9.59)	13.13 (12.21–14.12)	12.26 (11.35–13.23)	11.50 (10.57–12.49)
	>70 years#	7.54 (6.48–8.76)	12.12 (10.99–13.35)	13.16 (12.00–14.42)	9.85 (8.85–10.95)
**Marital status^abcd^**	Unmarried	4.72 (4.07–5.47)	9.35 (8.48–10.31)	8.19 (7.37–9.10)	7.37 (6.60–8.23)
	Married#	7.27 (6.73–7.85)	12.77 (12.14–13.43)	11.35 (10.72–12.01)	10.08 (9.46–10.74)
	Widow#	8.54 (7.15–10.16)	15.02 (13.41–16.79)	15.73 (14.07–17.54)	12.50 (11.10–14.06)
	Separated/divorced	8.07 (6.02–10.73)	15.55 (13.30–18.09)	13.4 5(11.38–15.82)	16.07 (13.37–19.20)
**Educational level^abcd^**	University	4.73 (3.82–5.85)	9.44 (8.38–10.62)	8.27 (7.26–9.42)	7.65 (6.59–8.88)
	Secondary#	5.02 (4.26–5.89)	9.95 (9.10–10.87)	9.99 (9.07–10.99)	8.24 (7.42–9.15)
	Primary	6.76 (6.20–7.36)	12.94 (12.20–13.72)	10.50 (9.79–11.25)	10.59 (9.87–11.36)
	No studies#	9.72 (8.49–11.12)	16.63 (15.03–18.36)	16.22 (14.87–17.66)	12.58 (11.24–14.05)
**Occupational status^abcd^**	Employed#	5.96 (5.46–6.49)	10.27 (9.62–10.96)	9.74 (9.11–10.40)	7.98 (7.33–8.68)
	Unemployed#	6.73 (4.77–9.40)	14.12 (12.15–16.36)	12.40 (10.88–14.10)	9.23 (8.00–10.62)
	Retired	7.66 (6.96–8.42)	13.62 (12.87–14.41)	12.03 (11.24–12.87)	11.75 (10.99–12.56)
**Immigrant^bcd^**	Yes	6.13 (4.01–9.26)	9.57 (7.92–11.52)	8.25 (6.73–10.09)	7.02 (5.55–8.85)
	No#	6.56 (6.15–6.99)	12.23 (11.73–12.75)	11.15 (10.66–11.66)	10.05 (9.57–10.56)
*Total prevalence of migraine* #		6.54 (6.14–6.97)	11.94 (11.45–12.44)	10.79 (10.31–11.29)	9.69 (9.22–10.18)

CI confidence Interval.

a Significant association for 2003 SNHS,

b Significant association for 2006 SNHS,

c Significant association for 2009 EHSS,

d Significant association for 2011 SNHS.

# Significant time trend from year 2003 to 2012 (P<0.05).

Prevalence of migraine was significantly higher in females than in males in every survey, showing an almost three time fold. The greatest crude values are observed in the 51–70 years age group in all surveys reaching the highest data within the 2006 SNHS (13.13%). Individuals with a marital status of “separated/divorced” or “widow” exhibited a higher prevalence of migraine than “unmarried”. We also found an inverse and significant relationship between educational level and the prevalence of migraine: those subjects with “no studies” were more likely to suffer migraine than those with “university studies”. Finally, subjects with an occupational status of “unemployed” or “retired” had higher prevalence of migraine when compared with the working population. In the last three surveys, immigrants exhibited lower prevalence of migraine than native adults.

Details of the prevalence of migraine according to lifestyle variables and self-perceived health are shown in **Table 3**. The bivariate analysis revealed that individuals who reported a health status of “fair/poor/very poor” were more likely to suffer from migraine (P<0.01). Individuals who sleep <8 hours/day and with a BMI >30 showed a higher prevalence of migraine than those sleeping ≥8 hours or with a BMI<30 (P<0.05). Non-smokers and individuals not drinking alcohol in the last 2 weeks were more likely to suffer from migraine (P<0.05). No significant differences between subjects practicing or not physical exercise exited in 2003, 2006 and 2009 (P>0.235); however, individuals not practicing physical exercise regularly (3 days/week) were more likely to suffer from migraine (P<0.05) in the last survey conducted in 2011–2012. Finally, as the number of chronic diseases increased, the prevalence of migraine also increased: subjects with ≥3 conditions exhibited a prevalence of migraine of 20% in all surveys.

**Table 3:** Prevalence of migraine by self-perceived health and lifestyle variables among Spanish adults, according to the national health surveys conducted in Spain from 2003 to 2012.

VARIABLE	CATEGORIES	2003 SNHS % (95%CI)	2006 SNHS % (95%CI)	2009EHSS % (95%CI)	2011 SNHS % (95%CI)
**Self-rated health^abcd^**	Excellent/good	3.83 (3.44–4.25)	7.65 (7.16–8.18)	6.91 (6.44–7.42)	6.51 (6.04–7.00)
	Fair/poor/very poor#	12.31 (11.39–13.29)	20.45 (19.44–21.51)	20.22 (19.12–21.36)	17.79 (16.69–18.95)
**Smoking habit^abcd^**	Smoker	5.38 (4.72–6.11)	11.82 (10.90–12.80)	9.38 (8.56–10.28)	9.86 (8.91–10.89)
	Ex-smoker	6.34 (5.44–7.38)	9.77 (8.84–10.78)	9.23 (8.29–10.27)	8.21 (7.32–9.19)
	Non-smoker#	7.31 (6.73–7.94)	12.89 (12.20–13.62)	11.98 (11.26–12.73)	10.17 (9.53–10.85)
**Alcohol consumption^abcd^**	No#	7.83 (7.18–8.54)	15.39 (14.59–16.24)	13.95 (13.12–14.83)	11.67 (10.96–12.42)
	Yes#	5.53 (5.04–6.07)	9.19 (8.61–9.80)	8.82 (8.26–9.42)	7.77 (7.17–8.42)
**Sleep habits^abcd^**	≥8 hours/night#	5.53 (5.00–6.11)	10.20 (9.53–10.91)	NA	7.84 (7.23–8.50)
	<8 hours/night#	7.59 (7.00–8.24)	13.56 (12.88–14.28)	NA	11.54 (10.85–12.27)
**Physical exercise** d	No	6.73 (6.20–7.30)	12.52 (11.73–13.35)	10.66 (10.03–11.33)	10.99 (10.25–11.76)
	Yes#	6.28 (5.68–6.94)	11.66 (11.04–12.32)	10.93 (10.23–11.67)	8.65 (8.06–9.28)
**Obesity^abcd^**	BMI<30#	6.26 (5.82–6.72)	11.39 (10.85–11.96)	10.22 (9.69–10.77)	9.08 (8.56–9.63)
	BMI≥30	9.06 (7.87–10.42)	14.01 (12.63–15.51)	13.06 (11.78–14.46)	11.22 (9.99–12.58)
**Number chronic conditions^abcd^**	None#	4.56 (4.14–5.03)	8.62 (8.06–9.22)	8.86 (8.33–9.42)	6.84 (6.32–7.40)
	One or two#	9.72 (8.85–10.65)	16.10 (15.19–17.05)	14.67 (13.70–15.70)	13.26 (12.35–14.23)
	Three or more	19.51 (16.53–22.86)	21.62 (19.35–24.07)	25.14 (21.01–29.77)	20.58 (18.20–23.18)

NA Not available: CI confidence Interval.

a Significant association for 2003 SNHS,

b Significant association for 2006 SNHS,

c Significant association for 2009 EHSS,

d Significant association for 2011 SNHS.

# Significant time trend from year 2003 to 2012 (P<0.05).

### Multivariate analysis

The time trend analysis revealed that the probability of suffering migraine over the last 12 months increased from 2003 to 2012 (adj. OR 1.65, 95%CI 1.50–1.81), being this increased higher in women (adj. OR 1.77; 95%CI 1.59–1.97) than for men (adj. OR 1.31; 95%CI 1.09–1.57).

The multivariate analysis separately for men and women are detailed in **Table 4**. After adjustment for all the covariates, the probability of Spanish women of suffering migraine was 3 times higher than for men (adj.OR 3.08, 95%CI 2.82–3.37). The age group with higher adjusted OR for migraine was the 31–50 years for the total population whereas the group aged >70 years showed the lowest adjusted OR in all surveys: data suggest a declining trend in migraine prevalence as the age rises (adj. OR 0.42, 95%CI 0.35–0.51).

**Table 4:** Variables significantly associated with a higher likelihood of suffering migraine among adults, according the national health surveys conducted in Spain from 2003 to 2012 and according to gender.

VARIABLE	CATEGORIES	MEN Adjusted OR 95% CI	WOMEN Adjusted OR 95% CI
**Age**	16–30 years	1	1
	31–50 years	0.79 (0.62–1.02)	1.20 (1.01–1.43)
	51–70 years	0.51 (0.39–0.67)	0.86 (0.71–1.05)
	>70 years	0.36 (0.27–0.50)	0.46 (0.37–0.58)
**Educational level**	University	1	1
	Secondary	1.10 (1.01–1.25)	1.16 (0.98–1.35)
	Primary	1.15 (0.96–1.19)	1.16 (0.99–1.35)
	No studies	1.28 (1.08–1.75)	1.34 (1.12–1.61)
**Immigrant**	Yes	1	1
	No	1.80 (1.23–2.64)	1.24 (1.01–1.52)
**Self-rated health**	Excellent/good	1	1
	Fair/poor/very poor	3.22 (2.68–3.98)	2.73 (2.47–3.02)
**Number chronic conditions**	None	1	1
	One or two	1.68 (1.40–2.04)	1.53 (1.37–1.70)
	Three or more	3.05 (2.18–4.27)	2.25 (1.86–2.73)
**Year of survey**	2003	1	1
	2006	1.63 (1.37–1.94)	2.02 (1.83–2.23)
	2009	1.54 (1.29–1.83)	2.17 (1.96–2.40)
	2011	1.31 (1.09–1.57)	1.77 (1.59–1.97)

The socio-demographic variables that were significantly associated with a higher likelihood of suffering migraine in Spanish adult besides gender were lower educational level (adj.OR 1.32, 95%CI 1.13–1.54) and not being an immigrant (adj.OR 1.37, 95%CI 1.15–1.64). A worse self-rated health status was related to higher prevalence of migraine for men (adj. OR 3.22; 2.68–3.98) and women (adj.OR 2.73; 2.47–3.02) The adjusted OR for suffering migraine increased as the number of chronic conditions raised, with the highest value being found for men who had 3.05 times more probability if they suffered ≥3 conditions compared with those men who had none.

## Discussion

The current study found that the prevalence of migraine has increased in the first decade of the 21^st^ century in Spain. In addition, migraine was also associated with being female, mid-age, low educational level, not being an immigrant, worse self-perceived health status and the presence of comorbid chronic conditions.

To our knowledge, this is the first national time trend estimation of prevalence of migraine in the Spanish adult population. The 1-year prevalence rates observed in our study were somewhat lower on 2003, but similar in 2006, compared to prevalence rates previously reported in other countries [2]–[8]. Differences between studies may be related to specific questions related to diagnosis of migraine, the nature of the national health care system, the role of pain and suffering in the culture, the number of compensations and law insurances, and the differences in biology between communities. In the current study, we considered migraine sufferers those subjects who answered “yes” to having migraine over the last 12 months and being diagnosed by a neurologist. Nevertheless, although neurologists usually use the International Headache Society criteria for making the diagnosis of migraine, the criteria were not incorporated in the main surveys. Thus, although questions regarding migraine presume that the condition should be diagnosed by a neurologist; there remained the potential for subjects to over- or under-report their pain and therefore, they did not attend to the neurologist. It is possible that point-time prevalence data of migraine are underestimated in the current study explaining the lower prevalence rate observed in 2003.

Our study observed an increase in the prevalence of migraine in the first decade of the 21^st^ century in Spain. Lyngberg et al found that migraine prevalence in Denmark slightly increased (11–15%), but not significantly, in the last decade of the 20^th^ century, although the proportion of individuals with migraine 14 days or more per year increased from 12% to 38% [12]. On the contrary, Linde et al recently observed a significant increase of the 1-year prevalence of migraine from 12.1% to 13.2% in the last years in Norway [13]. It is interesting to note that both studies show an increased temporal trend of the prevalence of migraine, although statistical significance was different. In fact, discrepancies in time trends are also reported in the prevalence of chronic pain in general since some authors found that the prevalence of chronic musculoskeletal pain increased in the last years [18] but others had reported that the prevalence of chronic pain remained stable [19], [20]. It is probable that different methodologies of collecting data can affect the results. Nevertheless, current results suggest that there is a trend showing an increase in the prevalence of migraine in Spain in the last years, at least in the 21^st^ century.

To determine plausible explanations for the increase in migraine prevalence is beyond the scope of this study; however, some factors are discussed. It is possible that changes in modern society including changes in social environments, sedentary life [21] higher stress [22], or unhealthier lifestyle habits [23] can be related to greater prevalence of migraine. It is also plausible that better application of diagnostic criteria in the last years tends to a better identification of this condition. Future studies should assess the potential risk factors associated with the increased prevalence of migraine.

The current study also found that migraine showed higher prevalence in women, mid-aged people, those with lower educational level and native Spaniards. These data are consistent with previous studies showing that migraine exhibits a peak prevalence between 25–50 years [2]–[6], [24]. Further, previous studies also support that the prevalence of migraine is higher in individuals with lower educational level [25], [26] suggesting a social causation hypothesis [27]. Finally the presence of higher prevalence of diagnosed migraine in native people may be related to a lower use of health care services by the immigrants [28].

It is known that individuals suffering migraine exhibit a detriment of quality of life [29]. We found that worse self-perceived health status and the presence of comorbid chronic conditions were also associated with migraine. In fact there is evidence showing that migraine is usually comorbid with chronic diseases [30]. Several theories are plausible for explaining the comorbidity of several conditions with migraine, but reviewing them are beyond the scope of this study. Therefore, it is possible that the presence of myriad of symptoms reduce the self-reported health status of the individuals. Further, clinicians should be aware of the comorbid conditions to improve the management of individuals with migraine.

Although strengths of our study include large sample sizes, a randomly selected sample of the population, the employment of standardized surveys, and training of the data collectors; there are also some possible limitations. First, it is difficult to determine temporal trends since even small differences in the methods can influence the results. In our study, we used 4 national surveys conducted during a decade including identical questions for increasing the feasibility. Nevertheless, although the validity of the questions included in the surveys to classify individuals as migraine sufferers has not been evaluated, the use of single questions for determining the presence of migraine is supported [31]. Thus, although questions regarding migraine presumed that the condition should be diagnosed by a neurologist, there remains the potential for subjects to over- or under- report their pain. However, our study follows the recommendations made by Lipton et al who found that to estimate migraine prevalence it is required that subjects reporting the disease have been also diagnosed by a neurologist [32]. Second, we did not assess the severity, pain-related disability, duration (acute, chronic) of migraine. Third, since we used data from cross-sectional surveys, we cannot determine potential cause-effect relationships determining the cause of time trends. Finally, information obtained on interviews is subjective and may be subject to recall errors or a tendency of subjects to give socially desirable responses in the interviews, particularly those regarding their lifestyle habits. Nevertheless, health surveys are significantly commended as they can collect valuable information related to health problems, which is not available from most other sources of information. In addition, population-based surveys similar to the used in our study have been employed by other authors in different countries to estimate the prevalence of migraine and related-factors. Despite these limitations our findings provide additional insight into demographic aspects of migraine in Spanish adults for which there is little information at a population level.

## Conclusion

This study found that the prevalence of migraine has increased in the first decade of the 21^st^ century in Spain. Further, migraine was also associated with being female, mid-aged, low educational level, not being an immigrant, worse self-perceived health status and the presence of comorbid chronic conditions. Future studies should investigate the cause of these changes in time trends for migraine.
